# Nutritional Strategies for Chronic Craniofacial Pain and Temporomandibular Disorders: Current Clinical and Preclinical Insights

**DOI:** 10.3390/nu16172868

**Published:** 2024-08-27

**Authors:** Kajita Piriyaprasath, Yoshito Kakihara, Mana Hasegawa, Yuya Iwamoto, Yoko Hasegawa, Noritaka Fujii, Kensuke Yamamura, Keiichiro Okamoto

**Affiliations:** 1 Department of Restorative Dentistry, Faculty of Dentistry, Naresuan University, Phitsanulok 650000, Thailand; kajitap@nu.ac.th; 2 Division of Oral Physiology, Faculty of Dentistry, Niigata University Graduate School of Medical and Dental Sciences, Niigata 951-8514, Japanyiwa@dent.niigata-u.ac.jp (Y.I.);; 3 Division of Dental Pharmacology, Faculty of Dentistry, Niigata University Graduate School of Medical and Dental Sciences, Niigata 951-8514, Japan; 4 Sakeology Center, Niigata University, Niigata 951-8514, Japan; 5 Division of General Dentistry and Dental Clinical Education Unit, Niigata University Medical and Dental Hospital, Niigata 951-8514, Japan; 6 Division of Dental Clinical Education, Faculty of Dentistry, Niigata University Graduate School of Medical and Dental Sciences, Niigata 951-8514, Japan; 7 Division of Comprehensive Prosthodontics, Faculty of Dentistry, Niigata University Graduate School of Medical and Dental Sciences, Niigata 951-8514, Japan; cem17150@dent.niigata-u.ac.jp

**Keywords:** chronic pain, diet, nutrition, nutraceuticals, temporomandibular disorders

## Abstract

This narrative review provides an overview of current knowledge on the impact of nutritional strategies on chronic craniofacial pain associated with temporomandibular disorders (TMDs). Individuals experiencing painful TMDs alter their dietary habits, avoiding certain foods, possibly due to chewing difficulties, which might lead to nutrient deficiencies. Our literature investigation revealed that the causal links between nutritional changes and craniofacial pain remain unclear. However, clinical and preclinical studies suggest that nutraceuticals, including vitamins, minerals, polyphenols, omega-3 fatty acids, isoprenoids, carotenoids, lectins, polysaccharides, glucosamines, and palmitoylethanolamides, could have beneficial effects on managing TMDs. This is described in 12 clinical and 38 preclinical articles since 2000. Clinical articles discussed the roles of vitamins, minerals, glucosamine, and palmitoylethanolamides. The other nutraceuticals were assessed solely in preclinical studies, using TMD models, mostly craniofacial inflammatory rodents, with 36 of the 38 articles published since 2013. Our investigation indicates that current evidence is insufficient to assess the efficacy of these nutraceuticals. However, the existing data suggest potential for therapeutic intervention in TMDs. Further support from longitudinal and randomized controlled studies and well-designed preclinical investigations is necessary to evaluate the efficacy of each nutraceutical intervention and understand their underlying mechanisms in TMDs.

## 1. Introduction

Chronic temporomandibular disorders (TMDs) present a range of conditions affecting deep craniofacial tissues, including the temporomandibular joint (TMJ) and jaw muscles [1]. These disorders are the second most prevalent musculoskeletal chronic pain condition [2]. Traditional medical strategies often rely on pharmacological interventions for chronic TMD pain management, which vary in efficacy and carry the risk of adverse effects [3,4]. Notably, guidelines from the American College of Physicians suggest that drugs might not be the first-line treatment for chronic pain [5]. In recent decades, there has been a growing body of evidence highlighting the significance of complementary and integrative approaches in managing chronic pain [6,7,8,9,10,11]. These approaches generally involve a diverse range of treatments categorized by their delivery modes, including mind–body therapy (e.g., massage, mindfulness), bioenergetic therapy (e.g., acupuncture) [10,12], and biologically based therapy (e.g., nutrition).

Among these modalities, dietary patterns have emerged as significant health influencers, playing critical roles in managing chronic diseases, including chronic pain [13,14]. Prioritizing a healthy diet and nutritional interventions remains crucial among modifiable behaviors. Addressing this complexity necessitates a comprehensive examination of how nutrients function within the body, particularly in the context of managing chronic pain.

Poor dietary choices, often associated with a Western diet marked by excessive consumption of processed foods, too much added salt, sugar, and unhealthy fats, have been linked to various chronic pain conditions [15]. For example, an energy-dense diet characterized by a high sugar intake has been positively correlated with chronic back pain [16]. Individuals exhibiting dietary patterns similar to those of the Western diet are more prone to developing rheumatoid arthritis [17], with high-sugar diets suspected to contribute to its onset [18]. Additionally, increased dietary salt intake has been associated with non-specific low back pain, possibly due to posterior lumbar subcutaneous edema [19]. Cross-sectional surveys indicate that red meat, fish, and legumes are frequently reported to exacerbate joint pain in rheumatic diseases [20], while excessive consumption of cured meats and sweetened beverages has been linked to increased psychological distress in fibromyalgia patients [21]. Similarly, obesity and elevated body fat content are documented to heighten the risk of fibromyalgia [22]. Furthermore, a high-sucrose diet has been found to expedite arthritis progression in rheumatoid arthritis models [23].

In contrast to the preceding discussion, dietary modifications (e.g., a plant-based diet) and specific nutritional approaches offer significant benefits in promoting health and alleviating chronic pain [24,25,26,27]. Furthermore, it has been demonstrated that a range of constituents in foods can modulate neural functions associated with chronic pain [26,27,28,29]. Hence, it is reasonable to hypothesize that alternative nutritional factors are pivotal in controlling chronic TMD pain. 

Given these considerations, although evidence regarding the effects of nutritional approaches on chronic deep craniofacial pain seen in TMDs is limited, several reports suggest that such approaches could significantly alleviate painful TMDs [30,31,32]. This narrative review article aims to assess the current understanding of the effectiveness of dietary habits and nutritional approaches for managing painful TMDs. It seeks to explore studies from the past 25 years and provide an updated investigation into the potential impact of nutritional approaches on TMDs in both clinical and preclinical settings. Due to existing evidence, there is particular emphasis on elucidating the effects of specific nutritional compounds, as shown in Table 1. Importantly, the focus is on compounds derived from ordinary foods such as vegetables, fruits, and fish. Further, given the scarcity of clinical evidence to evaluate such hypotheses, exploring preclinical research, particularly in preclinical models, could provide valuable insights into the effects of nutritional interventions on deep craniofacial nociception [33,34]. 

## 2. Methods

### 2.1. Search Strategy

This review article adheres to the rigorous guidelines set by the Scale for the Assessment of Narrative Review Articles [35]. The exhaustive search for relevant clinical articles was meticulously conducted using mainly Med-line PubMed, targeting keywords outlined in Table 2. Only articles written in English and published between January 2000 and March 2024 were primarily considered for inclusion, with strict exclusion criteria applied to unpublished results, proceedings papers, personal communications, and conference reports. During the backward search process, we identified significant papers published before 2000 or not found in PubMed, which were deemed relevant and therefore included for analysis. This article primarily focuses on clinical and preclinical evidence highlighting the nutritional roles in chronic pain associated with TMDs, occasionally comparing these findings with other chronic pain conditions. Particularly, in preclinical literature, evidence demonstrating nutritional roles in craniofacial nociceptive responses indicated by behavioral or neural responses is included for discussion. Thus, this narrative review article focuses less on the molecular mechanisms underlying painful TMDs and craniofacial nociception. Furthermore, it does not include reports in Table 2 from animal models mimicking pain conditions related to the trigeminal nerves, such as neuropathic pain, headaches, and toothaches. The screening process was conducted with utmost precision by three reviewers (K.P., Y.I., and M.H.), while the task of forward and backward searching was expertly executed by another two reviewers (K.O. and Y.K.). The eligibility of articles was carefully assessed by two supervisors (K.Y., and N.F.) to maintain the highest standards of quality and reliability. 

### 2.2. Quality of Evidence Assessments

Risk of bias was evaluated using the Cochrane Risk of Bias Tool for randomized controlled trials in clinical articles. Each item was categorized as low, high, or unclear risk of bias. For pre-clinical articles, the Systematic Review Centre for Laboratory Animal Experimentation (SYRCLE) criteria were used [36]. The assessment criteria were evaluated by three different authors (K.P., Y.H., and K.O.).

## 3. Results

### 3.1. Characteristics

#### 3.1.1. Clinical Articles

Twelve randomized controlled trials revealed the roles of nutraceuticals categorized into four groups on painful TMDs (Table 3). Five studies employed the oral administration of each nutraceutical solely, while seven studies employed the oral administration of each nutraceutical with additional interventions, such as hyaluronic acid injection into the TMJ [37,38,39,40,41,42,43]. Ten of these twelve studies focused on the effects of neutraceuticals on TMJ pain. One study examined the effects of minerals (magnesium) on myogenic types of TMDs. The remaining studies examined the effects of vitamin B12 on both muscle and TMJ pain.

#### 3.1.2. Preclinical Articles

Thirty-eight articles were assessed. Of these, thirty-three were conducted with rats, one with mice, and four with both mice and rats (Table 4). Two articles specifically employed female rats [49,50]. Effects of nutraceuticals on nociception were assessed through facial pain-like behaviors or neural activities evoked by craniofacial nociceptive stimulation. Models were primarily developed through craniofacial injections of inflammatory agents, including formalin, complete Freund’s adjuvant (CFA), mustard oil, capsaicin, and zymosan. Seven studies assessed nociceptive neural activities by quantifying the number of action potentials in the trigeminal ganglion or trigeminal subnucleus caudalis evoked by craniofacial stimulation [51,52,53,54,55,56,57]. Behavioral and neural responses were compared between groups treated with vehicle and nutraceutical.

### 3.2. Risk of Bias Assessment

#### 3.2.1. Clinical Articles

The risk of bias assessment for each article is described in detail in Table S1. No article was identified as having a high risk of bias.

#### 3.2.2. Preclinical Articles

The risk of bias assessment for each preclinical article is detailed descriptively in Table S2. Seven articles (18.4%) displayed a high risk of bias, while 33 articles (86.8%) had more than six out of ten items classified as “unclear”. In the text, no articles described the issues related to items, including the domain of allocation concealment, random housing, and random outcome assessment.

### 3.3. Effect of Interventions—Outcomes

#### 3.3.1. Clinical Articles

Ten out of twelve articles documented the beneficial effects of each nutraceutical in improving painful TMDs. However, two reported limited effects of them on osteoarthritis-type TMDs [39,47]. 

#### 3.3.2. Preclinical Articles

As shown in Table 4, all articles demonstrated inhibitory effects on craniofacial nociception indicated by decreases in pain-like behaviors or neural activities in response to craniofacial stimulation. The number of articles assessing each nutraceutical varied from one (palmitoylethanolamide) to twelve (polyphenol). Methodologically, different types of models with different pain assessments were conducted. Eighteen articles assessed the effects of nutraceuticals on acute facial pain-like behaviors evoked by formalin (twelve articles), capsaicin (two articles), or several agents (four articles). Twenty articles evaluated the effects of nutraceuticals on persistent facial pain-like behaviors in inflammatory pain models, such as CFA (ten articles), zymosan (four articles), and others. These variations in the assessment of each nutraceutical’s efficacy make it challenging to precisely analyze their beneficial effects on pain, including determining appropriate doses, sample sizes, treatment durations, and differences in efficacy between nutraceuticals. However, all articles revealed the inhibitory roles of nutraceuticals on craniofacial nociception.

## 4. Discussion

### 4.1. Characteristics of Dietary Habits and Nutritional Intakes in Painful TMDs

Recent research sheds light on the promising role of nutritional strategies in managing chronic pain conditions [87,88,89,90]. These approaches not only present a potentially cost-effective alternative to conventional medical treatments but also demonstrate a safer profile [91]. Moreover, they align with a patient-centered approach to treating chronic pain [8,92]. Emerging evidence suggests that specific dietary patterns exhibit beneficial effects on health promotion. For example, with a notable emphasis on a plant-based diet [26], recognized for its preventive and therapeutic impacts on diverse chronic diseases such as diabetes [93], cardiovascular diseases [94], and depression [95], the benefits extend to chronic pain as well. Notably, Mediterranean, vegetarian, vegan, or high-protein diets have shown efficacy in alleviating chronic pain associated with fibromyalgia [88,96], rheumatoid arthritis [97], musculoskeletal pain [98,99], low back pain [16], and headaches [100,101].

On the other hand, compared to the aforementioned chronic pain, the impact of dietary habits and regimens on TMDs, particularly the alleviating effects on painful TMDs, remains less clear. Before considering dietary and nutritional approaches for patients with TMDs, evaluating their nutritional status and habits is crucial. Furthermore, neurobiologically, the concepts discussed above, except headache, are based on chronic pain related to spinal pain mechanisms, while there remains uncertainty regarding their applicability to painful TMDs. Evidence indicates that TMDs involving trigeminal pain systems regulating deep craniofacial nociception exhibit distinct neural nociceptive features compared to spinal pain systems [1,33,102]. Thus, there is a need to assess the current knowledge of the nutritional roles in managing painful TMDs. In light of our above discussion, it is crucial to emphasize the distinctive attributes of TMDs, particularly their correlation with masticatory dysfunction and chronic pain in the deep craniofacial tissues. These factors exert a more pronounced influence on eating behaviors in TMD patients experiencing chronic pain compared to individuals with chronic pain in areas outside the orofacial region [103,104]. As a result, changes in dietary patterns, coupled with potential nutrient deficiencies, could lead to notable changes in trigeminal nociceptive mechanisms, potentially affecting the management of painful TMDs. Preclinical studies provide supporting evidence for this notion. For example, trigeminal inputs associated with chewing hard foods, as opposed to soft foods, have been implicated in reducing hindpaw nociception [105]. Furthermore, masticatory function might play inhibitory roles in nociception by activating descending pain controls [106], while dysfunctions in those controls are well documented in TMD patients [1,33]. This suggests that modifications in dietary behaviors, leading to changes in trigeminal systems, could induce functional alterations in the central nervous system beyond the trigeminal systems to some extent. Furthermore, these findings underscore the unique characteristics that differentiate the underlying mechanisms of TMDs from those of other pain conditions, particularly in terms of their nutritional implications. Hence, a possible link exists between painful TMDs and dietary modifications; however, they do not definitively establish a causal relationship. In other words, these observations could elucidate how TMDs influence dietary habits and masticatory function, yet they do not address the impact of specific dietary regimens or their constituents on TMD pain.

It is possible that alterations in dietary patterns and nutritional intake, potentially leading to deficiencies of specific nutrients, could contribute to the onset or aggravation of TMDs [32]. TMD patients often adapt their diets by cutting food into smaller pieces [103], and those experiencing chronic masticatory pain consume raw whole fruit less frequently [107]. Studies using the Test of Masticating and Swallowing Solids (TOMASS) reveal that TMD patients take longer to consume certain foods [108]. Reports indicate that TMD patients might eliminate specific foods like meat, apples, and bread, favoring softer cooking methods for vegetables and meat [109,110], potentially leading to deficiencies in certain nutrients. Furthermore, myogenic TMDs could be linked to reduced dietary fiber intake, which is prominently found in plant-based foods [111]. On the other hand, a recent study demonstrated that the macro-nutritional status—such as levels of energy, protein, carbohydrate, and fat—of individuals with TMDs is similar to that of healthy individuals, although this study evaluated only the amount of macronutrients using 24 h dietary recall methods [108]. While at this stage it is difficult to draw strong statements about the differences in dietary habits and nutritional status in daily life with painful TMDs, these findings suggest that micronutrients rather than macronutrients might be essential for regulating deep craniofacial nociception. The following section elucidates the existing knowledge regarding the impact of several nutrients on painful TMDs. It also examines preclinical evidence from animal models of deep craniofacial pain [33].

### 4.2. Nutraceutical, Nutrition, and Painful TMDs

Nutraceutical, a term commonly used, has attracted considerable interest among individuals aiming to self-manage their chronic pain using readily accessible supplements [27,112]. Several analogous nutraceutical terms are found, such as functional foods, nutritional, and dietary supplements. Currently, the definition of nutraceutical varies between researchers and even countries. Therefore, the distinction between these terms is beyond the scope of this article. Recent reports, including meta-analyses, have emphasized various nutraceuticals emerging as viable options for managing a wide range of chronic pain conditions, such as osteoarthritis [113], neuropathy [114], fibromyalgia [115], irritable bowel syndrome [116], and headache [117]. On the other hand, our literature search reveals less comprehensive elucidation on the roles of nutraceutical interventions in TMDs. As conclusive statements to draw the efficacy of each nutraceutical are premature due to limited evidence, we discuss articles that explore the role of nutraceuticals in chronic pain conditions, including TMDs, in the following sections.

#### 4.2.1. Vitamins

Vitamins are a group of organic compounds such as four fat-soluble vitamins (A, D, E, K) and nine water-soluble vitamins (B and C). Multiple clinical studies have revealed serum levels of specific vitamins and their supplementary impacts on chronic TMD pain. Our literature search found two clinical and two preclinical articles evaluating the direct effects of vitamins D and B12 on painful TMDs and craniofacial pain-like responses in preclinical models. Indeed, other studies indicated a significant possibility of the influence of vitamin D on TMDs. This focus is likely derived from the prevalence of articles that explore the correlation between vitamin D and bone metabolism in the TMJ but not pain, which is beyond our aims. Such findings are particularly relevant in the context of painful conditions like osteoarthritis [118,119,120,121,122,123]. Subsequently, we discussed the current knowledge of reports that assessed the roles of other vitamins, including vitamin B complex and vitamin C [124].

1. Vitamin D

Vitamin D deficiency has been implicated in the development of musculoskeletal disorders, leading to reduced bone density, muscle weakness, and chronic pain [125,126]. Several articles have suggested a potential association between vitamin D deficiency and an increased risk of developing painful TMDs [118,122]. However, research findings on its serum levels in TMD patients have been inconsistent. Some studies have demonstrated decreased levels [37,119,120], while others have found less significant change [121,127,128]. The reasons for these discrepancies are unclear; however, decreased levels of vitamin D have been observed in various types of TMDs, including intra-articular disorders [37,121] and extra-articular disorders such as myogenic TMDs [127]. These findings could be consistent with observations of vitamin D deficiency in other conditions, such as fibromyalgia [125] and osteoarthritis [129]. Furthermore, polymorphisms in the vitamin D receptor have been linked to disc degeneration-related pathologies, such as osteoarthritis [130]. However, their susceptibility to TMJ internal derangement, TMJ osteoarthritis, and pain remains uncertain [131]. These findings suggest that the development of painful TMDs associated with vitamin D is primarily attributed to the level of vitamin D itself rather than the processing of its ligand reception at least. Emerging reports have demonstrated the beneficial effects of vitamin D supplementation on various types of chronic pain. However, the findings have been inconsistent. For example, a previous cohort study and a recent systematic review have demonstrated that vitamin D supplementation significantly relieves chronic pain in conditions such as rheumatoid arthritis and fibromyalgia [132,133,134]. Conversely, other randomized controlled trials have found minimal effects of vitamin D supplementation on musculoskeletal pain [135]. Despite such inconsistencies, Lombardo et al. suggested that the potential benefits of vitamin D supplementation in alleviating chronic musculoskeletal pain and fibromyalgia might be observed in individuals deficient in vitamin D [134]. Consistent with the reports above, Kui et al. [122] indicated that patients suffering from TMDs with deficient levels of vitamin D are likely to benefit from vitamin D supplementation. A randomized controlled trial demonstrated that the beneficial effects of splint therapy combined with vitamin D supplementation on painful TMDs were evident in patients with lower serum levels of vitamin D [37].

2. Vitamin B complex and C

Our literature search revealed that one clinical and two preclinical articles investigated the influences of vitamin B on painful TMD and craniofacial nociception. No clinical and preclinical evidence evaluating the effects of Vitamin C on painful TMDs is found. Vitamin B complex (Bs) encompasses a group of eight essential water-soluble vitamins: B1 (thiamine), B2 (riboflavin), B3 (niacin), B5 (pantothenic acid), B6 (pyridoxine), B7 (biotin), B9 (folate), and B12 (cobalamin). We found the reports elucidating on the role of vitamin Bs, except vitamins B1, 3, 5, and 7, in nociception in clinical and preclinical settings of TMDs because those of other vitamin B have not been identified in our literature search. In the context of TMDs, clinical studies have shown variations in serum levels of these vitamins among patients. One study [123] found that TMD patients commonly exhibit deficiency in vitamin B12, while vitamin B9 levels remain unaffected. Conversely, another study [124] observed that TMD patients with a history of implant surgery showed deficiencies in vitamins B1, B6, and B12, as well as in vitamin C. However, those studies did not determine the association of chronic pain with the levels of each vitamin. Regarding other chronic pain conditions, a study reported a significant association between myofascial pain syndrome and reduced serum levels of vitamin B12, while levels of vitamin B9 remained unaffected [136]. Notably, the study also revealed a negative correlation between vitamin B12 levels and psychological distress, which plays a crucial role in exacerbating chronic pain [136]. These findings indicate the potential impact of vitamin B12 on myofascial pain syndrome and psychological distress. Additionally, in the cases of trigeminal pain systems, riboflavin (vitamin B2) and vitamin B6 have shown efficacy in preventing migraine headaches [137,138,139], and vitamins B6, B9 and B12 have been closely linked to secondary burning mouth syndrome [140]. Further, the web-based dietary search revealed that low levels of vitamin B12 intake might be associated with non-specific chronic pain [141]. Interestingly, while individuals are aware that vitamin B12 deficiency correlates with lower consumption, relying solely on meat is insufficient to prevent such deficiency [141]. It is well known that vitamins B6 and B12 are primarily obtained naturally through the consumption of various animal products, including meat, poultry, fish, and eggs, while vitamin B9 is found in dark green leafy vegetables (such as spinach and kale), legumes (like lentils and beans), citrus fruits and juices, avocado, and nuts.

Clinical research highlights the association between deficiencies in vitamin Bs and the development or alleviation of chronic pain conditions, particularly neuropathic pain [114,142,143,144,145]. Karaganis et al. commented that while most beneficial outcomes were reported against baseline measures, few positive comparisons were made against the placebo group [144]. Further, there is a report suggesting that vitamin B12 might not have beneficial effects on neuropathic pain [146], while methylcobalamin, the activated form of vitamin B12, could alleviate diabetic neuropathy, low back pain, and neuralgia [147]. The existing clinical literature concerning vitamin B supplementation’s efficacy in managing painful TMDs is somewhat scarce. However, one study has indicated that the use of vitamin B12 supplementation might enhance the inhibitory effects of photobiomodulation techniques, such as laser therapy and acupuncture, despite the study’s small sample size [38]. Consequently, preclinical studies could provide valuable insights into the potential contributions of vitamin B. One research group has delved into the effects of vitamin B12 on orofacial nociception through the orofacial formalin test in two separate reports [58,59]. In the first study, systemic administration of vitamin B12 demonstrated inhibition of orofacial formalin-induced nocifensive behaviors [58]. Notably, vitamin B12 exhibited enhanced antinociceptive effects when administered systemically alongside diclofenac. In the second study, intra-hippocampal administration of vitamin B12 also reduced orofacial nocifensive behaviors, potentially mediated by its involvement in opioid mechanisms [59]. Furthermore, despite the use of different orofacial pain models, consistent results have been observed regarding the beneficial effects of vitamin B, containing B1, B6, and B12, in alleviating the impacts on orofacial nocifensive behaviors in rats with chronic constriction injury of the infraorbital nerve [148].

Vitamin C, also known as ascorbic acid, is also a water-soluble vitamin that cannot be synthesized in the human body and is found prominently in fruits and vegetables. Clinical studies have demonstrated the association between vitamin C deficiency and chronic spinal pain conditions, including musculoskeletal pain, cancer-related pain, and orthopedic post-surgical pain [149]. In the case of TMDs, our literature search has not yielded reports demonstrating the impact of altered vitamin C levels on painful TMDs. This absence of findings could be interpreted in light of several observations. Firstly, vitamin C deficiency has been noted in elderly hospitalized patients, with trauma and surgery known to cause a significant reduction in serum vitamin C levels [150]. It is widely acknowledged that in many cases of TMDs, extensive surgery or obvious trauma is not apparent. Mehra et al. [124] reported vitamin C deficiency in TMD patients; however, these patients were not typical cases of TMDs, as they underwent implant surgery. Secondly, a retrospective study has revealed that a significant deficiency of serum vitamin C level was more likely to be seen in males than females [151], whereas TMD patients are well documented to be predominantly female [152]. Thirdly, although deficiency of vitamin C level (<11 µmol/L) is relatively rare in developed countries like the United States (6%) [153,154], it is unclear if this is the case for painful TMDs. As aforementioned, specific features of TMDs, like masticatory dysfunction, might hinder the intake of a vitamin C-rich diet, such as fruits and vegetables [107,109,110]. At present, the effects of vitamin C supplementation on TMDs remain unknown. However, there is substantial evidence suggesting that vitamin C effectively inhibits the response of various proinflammatory biomarkers, including interleukins and tumor necrosis factor, as well as counteracting oxidative stress conditions [29,149,155,156]. These factors are thought to be potentially associated with TMD pathology. Furthermore, vitamins C and D are also documented to have antioxidant and anti-inflammatory properties, capable of reducing reactive oxygen species production and inflammation [29]. Additionally, in cases of pain related to the trigeminal nervous system, supplementation with vitamin C has demonstrated efficacy in alleviating other forms of orofacial pain, such as pain induced by the third molar extraction [157]. 

#### 4.2.2. Minerals

The relationship between minerals and chronic pain varies depending on the specific minerals and types of chronic pain conditions involved. Several studies have examined these complexities within TMDs [119,123,124,128]. These investigations have primarily focused on serum levels of minerals such as magnesium (Mg), zinc (Zn), and calcium (Ca), yet their findings have shown inconsistencies. For instance, TMDs characterized by TMJ disk displacement with reduction exhibited similar levels of Mg compared to healthy controls [119,128], while TMDs following implant surgery displayed lower Mg levels than controls [124]. Similarly, while some studies reported lower levels of Ca in certain TMD cases [119], others found them comparable to healthy controls [128]. However, these studies overlooked myogenic types of TMDs, potentially limiting the scope of their conclusions. Additionally, they did not evaluate the correlation between these minerals and painful TMDs, further restricting the comprehensiveness of their findings. Furthermore, a cross-sectional study revealed that patients with TMDs displayed significantly lower serum levels of potassium despite being within the normal range compared to healthy controls. The association of potassium levels with painful TMDs remains unclear [123]. Potassium, primarily sourced from fruits and vegetables, might be challenging for TMD patients to consume, potentially correlating with TMD development. Despite limited evidence, several reports suggest that changes in mineral levels, possibly due to dietary customs and behaviors, might play a role in TMDs. These studies further highlight the potential roles of mineral supplementation in the treatment of painful TMDs. Our literature search based on one clinical and four preclinical articles demonstrated the inhibitory impacts of painful TMDs and craniofacial nociception; however, this current understanding might not allow us to provide strong evidence of the treatment efficacy of mineral interventions. In the following section, we discuss the involvement of magnesium, zinc, strontium, and sulfur in TMDs and craniofacial pain conditions, as several reports support the hypothesis of their relationship.

1. Magnesium (Mg)

Mg, as a known calcium channel blocker, has garnered significant attention for its potential to alleviate various neurological diseases, including chronic pain [158]. The Western-type diet contains less Mg, whereas green vegetables such as spinach are major sources of this mineral [159]. Studies indicate that Mg plays a protective role against excessive neural excitation, which can lead to excitotoxicity [159]. Accordingly, evidence indicates the alleviating effects of magnesium on conditions such as migraine [160], fibromyalgia [158], low back pain [161], and visceral pain [89]. Local administration of Mg sulfate could prevent spinal sensitization, as indicated by increased glial activation in a rat model of incisional pain [162].

Studies assessing the impact of mineral interventions on TMDs are limited; however, several reports suggested that dietary magnesium might play a role in painful TMDs. A randomized clinical study demonstrated that local injection of Mg is an effective treatment for myofascial trigger points of the masseter muscle, leading to pain reduction and improvements of maximum mouth opening [44]. Likewise, a preclinical study revealed that oral administration of Mg prevented the development of orofacial pain-like behaviors in TMJ-inflamed rats, mimicking the conditions of TMDs, and further, treatment with Mg resulted in a decrease in phosphorylation of NR1 protein, one of the subunits of NMDA receptors [60]. Of note, changes in neural mechanisms related to NMDA receptor-mediated nociceptive processing could be essential to increase deep craniofacial nociception [163,164]. Similarly, two preclinical studies demonstrated that systemic administration of Mg reduced formalin-evoked facial pain-like behaviors [61,62]. Conversely, Mg deficiency caused increases in spontaneous facial pain-like behaviors in TMJ arthritis rats [60].

2. Zinc (Zn)

Zn has been investigated for its potential in treating various chronic pain conditions and could reduce nociceptive responses in preclinical models, possibly due to its anti-inflammatory properties [114]. In cases of orofacial pain, Zn supplementation therapy could alleviate the burning sensation associated with Burning Mouth Syndrome (BMS) patients [165]. However, it is noteworthy that over 94% of examined BMS patients exhibited normal serum Zn levels [166]. Additionally, studies indicate that administering Zn did not ameliorate symptoms such as oral burning sensation and discomfort in patients with oral lichen planus [167]. Patients with chronic myofascial pain displayed normal and lower levels of Zn in blood serum and intracellular stores (in erythrocytes), respectively [168]. This discrepancy in Zn levels suggests a potential link between Zn metabolism and the development or exacerbation of chronic pain. Our literature search did not yield evidence supporting the supplemental effects of Zn on TMDs. Despite a report with a small sample size (n = 23), Zn deficiencies observed in TMDs suggest that Zn might impact the management of painful TMDs [124].

3. Strontium

Strontium is found naturally in many foods, such as fish, vegetables, grains, and dairy products. Evidence suggested that strontium could exhibit inhibitory impacts on chronic pain conditions, potentially due to the positive effects of bone metabolisms [169,170]. For example, strontium injection can relieve pain in patients with painful bone metastases in a previously irradiated site [171]. While the roles of strontium on painful TMDs are lacking, in preclinical studies using TMD pain, systemic administration of strontium ranelate, a drug usually prescribed to treat osteoporosis, has shown potential in reducing pain-like behaviors. Interestingly, the antinociceptive effects of strontium ranelate observed in the TMJ inflammatory pain model might be attributed to the reduction in inflammatory cytokines, such as TNF-alpha, rather than a decrease in local inflammation [63]. These findings highlight the potential for exploring the effects of strontium in managing craniofacial pain, including TMDs.

4. Sulfur

Sulfur, a mineral found in our bodies and available in our diets, is primarily derived from proteins in various foods [172]. Sulfur sources that could affect chronic diseases include sulfur amino acids, methylsulfonylmethane, sulfur dioxide, and hydrogen sulfide. While the roles of these sulfur compounds in chronic pain are unclear, several reports suggested potential insights. For example, sulfur amino acids like cysteine, as part of protein structures, act as antioxidative metabolic intermediates such as glutathione [172]. Interestingly, therapy using hot springs containing the radioactive gas radon has increased glutathione levels in the brain, providing protective antioxidative functions against brain injury [173]. These findings support the notion that sulfur amino acids might exert antinociceptive effects; however, evidence on pain relief in TMDs is lacking.

Methylsulfonylmethane (MSM) is a notable organic sulfur-containing compound widely used as a dietary supplement for various conditions, such as pain, inflammation, and arthritis [174]. A randomized controlled trial indicated that a 12-week MSM regimen could improve knee quality of life, including pain [175]. However, another randomized controlled trial found no significant difference between MSM supplementation and placebo for knee osteoarthritis pain [176]. These inconsistent findings might be due to differences in pathologies of chronic pain. In the case of TMDs, one report revealed that MSM supplementation could have less beneficial effects on pain in osteoarthritis of the TMJ [39]. 

#### 4.2.3. Polyunsaturated Fatty Acids

Fatty acids are categorized depending on their length and degree of saturation into saturated fatty acids (FAs), monounsaturated FAs, and polyunsaturated FAs. Polyunsaturated FAs are divided into omega-3 and omega-6 FAs [177]. These FAs are indispensable for human health and show promise as natural remedies for chronic disorders [178,179,180], which are abundantly found in certain fish, nuts, and seeds.

Research has highlighted the contrasting roles of omega-6 and omega-3 FAs [181]. Notably, omega-3 FAs have been shown to alleviate chronic pain across various conditions, including musculoskeletal pain [27,98], low back pain [182], osteoarthritis [183], rheumatoid arthritis [184], and headaches [185]. Moreover, several studies have investigated the potential causal relationship between omega-3 FAs and chronic pain, revealing that increased circulating omega-3 FA levels might reduce the risk of low back pain [186] and pelvic pain [187]. Consistent with those clinical reports, omega-3 FAs exerted antinociceptive effects in various preclinical models [188,189]. Additionally, eicosapentaenoic acid (EPA) and docosahexaenoic acid (DHA), major polyunsaturated FAs, can act as substrates for the synthesis of specialized pro-resolving lipid mediators (SPMs) like resolvins, protectins, and maresins [190]. While these SPMs play a pivotal role in resolving inflammation by facilitating the clearance of inflammatory cells and debris, resolvin D1 could decrease hyperexcitability of nociceptive neurons in the trigeminal subnucleus caudalis in the facial inflammation rats [191].

In contrast, omega-6 FAs, enriched in the Western-style diet, are often regarded as detrimental to health promotion due to their potential to elevate levels of various proinflammatory substances such as prostaglandins, inflammatory cytokines, and inflammatory eicosanoids [192]. A randomized clinical trial with a diet low in omega-6 FAs and high in omega-3 FAs displayed decreased pain frequency and intensity in chronic headache patients [193]. A cross-sectional study found that a lower omega-6 to omega-3 FA ratio is linked to clinical and experimental pain responses in knee pain [194]. Recently, Sanders et al. suggested that omega-6 FAs could promote a generalized upregulation of nociceptive processing. They showed that omega-6 FAs were negatively associated with lower mechanical nociceptive thresholds in nociplastic pain conditions [182]. Preclinical studies supported the pronociceptive roles of nociception. Boyd et al. demonstrated that omega-6 FA intakes could induce persistent mechanical and thermal nociceptive hypersensitivity in the hindpaw due to neural changes in primary afferent nerves associated with elevated phospholipase A2-mediated lipid release. Of interest, pronociceptive changes could be rescued by intakes of omega-3 FAs [195].

Consequently, this article will delve into the influence of omega-3 FAs on painful TMDs. The physiological mechanisms of omega-3 FAs have been intensively investigated from a broad perspective, and their safety has been reported [177]. Currently, there is limited research exploring the roles of omega-3 FAs on TMDs; however, two cross-sectional studies have suggested an association between a higher ratio of omega-6 to omega-3 FAs and painful TMDs [196]. Similarly, a higher level of circulating omega-3 FAs has been linked to a reduced likelihood of painful TMDs [197]. Further, considering that psychological distress is a risk factor for TMDs, the preventive effects of omega-3 FAs on depression may support the idea of their inhibitory role in TMDs [178].

Preclinical studies have provided interesting evidence supporting the effectiveness of omega-3 FAs in managing TMJ inflammation. For example, Marana et al. [49] demonstrated that systemic administration of omega-3 FAs led to a decrease in TMJ damage and reduced levels of proinflammatory cytokines, such as IL-1 beta and TNF-alpha, in rats subjected to ovariectomy and rheumatoid arthritis induction. Similarly, systemic administration of omega-3 FAs exhibited anti-inflammatory effects, as evidenced by decreased levels of various proinflammatory cytokines in facial inflammatory models [64,65]. These findings highlight the potential of omega-3 FAs to inhibit local inflammation within the TMJ.

On the other hand, regarding the clinical features of TMDs, it is important to note that TMDs often present with fewer obvious inflammatory signs in craniofacial tissues. In this context, the potential of omega-3 FAs to alleviate craniofacial pain in TMDs might be mediated by mechanisms within the central rather than the peripheral nervous system. This idea finds support in several studies. For example, preclinical studies have shown that omega-3 FA deficiencies can induce emotional and neural disturbances similar to those observed after social defeat stress conditioning [198]. Consistently, supplementation with omega-3 FAs has been documented to have antidepressant effects [199,200]. Furthermore, a neuropathic pain model demonstrated hyperalgesia-like responses alongside decreases in DHA levels, while central administration of DHA decreased central microglia-associated neuroinflammation and pain-like behaviors [201]. In an intermittent cold stress model, omega-3 FAs exhibited modulatory effects on neuroinflammation related to transient receptor potential V1 signaling in the prefrontal cortex, hippocampus, and periaqueductal gray [202]. Finally, in the case of TMD models, systemic administration of DHA exerted inhibitory effects on neural activities in the trigeminal subnucleus caudalis in the persistent facial inflammatory pain rat [66].

While neural function heavily depends on adequate omega-3 FA levels, the precise mechanisms by which omega-3 fatty acids exert inhibitory effects on nociception remain unclear. Several findings might partially explain them [200,203]. For example, the facilitative effects of EPA and DHA on microglial autophagy might reduce inflammatory processes [204,205], leading to decreased nociceptive responses in the brain. Despite variations in chronic pain conditions, omega-3 FAs could play beneficial roles in alleviating deep craniofacial nociception. Further clinical and preclinical investigations into their effects on TMDs are warranted.

#### 4.2.4. Polyphenols

Polyphenols are natural compounds with phenolic structures found in various foods, such as fruits. Clinical and preclinical studies have evaluated the therapeutic roles of polyphenols on chronic pain. Accordingly, randomized controlled trials have shown that several fruits, such as blueberries [206], passionfruit [207], and strawberries [208], could offer relief for musculoskeletal pain, likely due to the antioxidative effects of various polyphenols that they contain. A meta-analysis demonstrated the beneficial roles of these for fibromyalgia patients [209]. Similarly, consuming fruits and vegetables has been linked to reduced odds of headaches in university students [210]. Polyphenol-rich foods have also been shown to reduce chronic pain in women with fibromyalgia [211]. Further, evidence has revealed that various polyphenols play beneficial roles in improving rheumatoid arthritis [184], irritable bowel syndrome [89], and migraine headaches [117].

While our literature search yields fewer randomized controlled studies assessing the effects of polyphenols on painful TMDs, a report not cited in PubMed has elucidated their beneficial roles in TMDs [212]. Moreover, one randomized control trial revealed the beneficial roles of avocado–soybean unsaponifiable extract that could contain polyphenols [213]. Additionally, several studies have shown that TMD patients experience alterations in oxidative status within the TMJ disc [156,214,215]. These findings suggest that targeting oxidative conditions with polyphenols could help reduce painful TMDs. Despite the limited clinical evidence, accumulating studies emphasize the effects of polyphenols on pain-like behaviors and their neural mechanisms in preclinical models of TMDs. For example, extracts rich in polyphenols from grape seeds [216] and purple corn [217] have shown promise in reducing neuronal and glial responses in critical areas associated with craniofacial nociception. The latter study highlighted the polyphenol content in purple corn extracts [217]. Similarly, citrus fruits [71,72] and cocoa [218,219], which also contain polyphenols, have demonstrated potential antinociceptive properties in facial pain models. 

A randomized experimental clinical study revealed that intake of chocolate, a cocoa-derived product rich in polyphenols, could reduce pain sensation caused by the injection of hypertonic saline into the masseter muscle in healthy individuals; however, the authors did not specifically assess the role of polyphenols in the observed reduction in pain responses [220]. An in vitro study demonstrated that cocoa bean extracts containing polyphenols could repress the release of calcitonin gene-related peptides in the trigeminal root ganglion [221], potentially regulating craniofacial nociception. Consistently, repeated administration of the coffee polyphenolic extract (CE) modulated reflexive pain responses, depressive-like behavior, and spinal cord gliosis in a dose-dependent manner in a model of fibromyalgia [222]. These effects are believed to arise from the polyphenols, which impact neural changes associated with orofacial nociceptive processing [29]. In the following paragraph, we demonstrate several preclinical examples of the roles of polyphenols, including quercetin, (-)-epigallocatechin-3-gallate, resveratrol, and curcumin on facial pain because of the available evidence regarding polyphenols.

Local administration of quercetin, one of the flavonoid phytochemicals, exerted inhibitory effects on nociceptive neural activities evoked by facial stimulation in trigeminal primary sensory neurons in the presence [51] and absence of persistent facial inflammatory conditions [52]. Further, (-)-epigallocatechin-3-gallate, the main catechin contained in green tea, could inhibit nociceptive neural activities in the trigeminal subnucleus caudalis region [55]. Likewise, daily administration of resveratrol contained abundantly in grapes and wine exerted inhibitory impacts on the trigeminal nociception in response to facial stimulation [56,223]. Notably, Ma et al. illustrated that the development of TMJ inflammation, accompanied by increased neural activities in the trigeminal subnucleus caudalis region due to microbiome perturbation, could be mitigated by systemic administration of resveratrol [67]. These findings suggest that the involvement of polyphenols in gut microbiota could have beneficial roles in alleviating deep craniofacial pain. Moreover, curcumin, a naturally occurring polyphenolic compound found in turmeric, exhibits antioxidative and anti-inflammatory properties. Systemic administration of curcumin inhibited pain-like behaviors in the hindpaw [224], trigeminal neuralgia model [225], and orofacial formalin pain model [50]. An in vitro study revealed that curcumin can inhibit key molecular processes in TMJ inflammatory chondrocytes [226], suggesting the antinociceptive roles of curcumin [227]. 

Collectively, while twelve preclinical studies suggest promising therapeutic roles for polyphenols in managing painful TMDs, further longitudinal and randomized controlled trials are needed to confirm their potential benefits, and the clinical efficacy of polyphenols is unclear.

#### 4.2.5. Isoprenoids

Isoprenoids are found in various common foods such as fruits, vegetables, nuts, and seeds. They are a class of natural compounds, including terpenes, that could exert antioxidative potential and help in pain relief [228]. Although clinical evidence regarding the roles of isoprenoids in TMDs is lacking, preclinical evidence supports their antinociceptive properties. For example, limonene, a monoterpene found in citrus fruits like lemons and oranges, has been shown to inhibit TMJ nociceptive behavior and alter neural responses in the trigeminal subnucleus caudalis [71]. Similarly, limonene’s inhibitory effects on nociception have been observed in models of fibromyalgia [229] and neuropathic pain [230]. Furthermore, systemic administration of citral, another monoterpene, has been found to attenuate masseter muscle pain-like behaviors induced by various algesic and inflammatory agents through the regulation of several transient receptor potential channels [73]. Prophylactic and therapeutic administration of citral also inhibited mechanical and heat hypersensitivity of the facial skin in rats with persistent TMJ inflammation [72]. Those findings from craniofacial pain models are consistent with those seen in hindpaw inflammatory, neuropathic, and plantar incisional pain models [231]. Additionally, carvacrol, another type of isoprenoid, has exerted antinociceptive effects in craniofacial pain models [232]. However, that isoprenoid was derived from aromatic and medicinal plants, not from ordinary foods. Based on three preclinical studies and the absence of clinical evidence, it is still too early to make definitive conclusions. However, it is worth considering that isoprenoids might have therapeutic potential in treating painful TMDs. Consequently, the efficacy of isoprenoids for managing painful TMDs remains uncertain.

#### 4.2.6. Carotenoids

Carotenoids are naturally occurring pigments synthesized by plants like carrots, sweet potatoes, avocados, and algae [233]. More than 1000 natural carotenoids have been documented, and only 40–50 of them are consumed in human diets, such as lutein and beta-carotene. They are responsible for the bright red, orange, and yellow colors of many fruits and vegetables and exert critical roles in human health as antioxidant properties by inhibiting reactive oxygen reactions and sources of vitamin A [234]. Evidence revealed that carotenoids could offer protection against local inflammation associated with rheumatoid arthritis [90]. For example, two preclinical studies have demonstrated the analgesic effects of crocin, a compound found in saffron and gardenia flowers, often used as a flavor. In one study, intra-cerebroventricular administration of crocin reduced pain-like behaviors in sleep-deprived rats [235]. In a relevant context to craniofacial pain, crocin inhibited facial pain-like behaviors triggered by formalin injection in rats [74]. Lutein is the most widespread carotenoid found in fruits and leafy vegetables. Preclinical reports demonstrated that lutein administration could exert inhibitory effects on various pain conditions. For example, lutein could potentially promote antioxidative, antidepressant, and antinociceptive effects in the preclinical models of fibromyalgia [236]. Regarding the conditions of facial pain, two reports revealed that lutein decreased nociceptive neural activities indicated by c-Fos expressions [75] and neural activities [76] in the medullary and upper cervical dorsal horn junction region. Further clinical and preclinical investigations would be needed to confirm and expand upon these findings regarding the potential roles of carotenoids on painful TMDs. Thus, the efficacy of carotenoids on painful TMDs is unclear.

#### 4.2.7. Lectin

Lectins are a heterogeneous group of proteins found in both plants and animals that can bind to carbohydrate molecules. It is well known that seaweed and beans are major sources of lectins, which exhibit antinociceptive and anti-inflammatory properties [237,238]. The biological activities of lectins might be related to their ability to recognize carbohydrates on the surface of cell membranes and trigger intracellular signaling that results in biological responses, including nociceptive processing. Clinical evaluations of the therapeutic roles of lectins in painful TMDs are lacking, yet preclinical studies have shown that lectins derived from seaweed [77,78] and seeds [79,80,81,82] could reduce facial nociception and inflammation in craniofacial tissues by regulating molecular mechanisms in TMD models. While the involvement of lectins in nociception is not fully understood, evidence suggests that plant lectins can modulate transient receptor potential vanilloid one function, leading to a reduction in formalin-evoked facial pain-like behaviors [78]. Additionally, the inhibitory effects of lectins on TMJ pain-like behaviors and inflammation could be due to decreased levels of proinflammatory cytokines, such as interleukin-1 beta, in TMJ tissues and the trigeminal ganglion [81]. Because no clinical evidence is available, further clinical and preclinical evaluations are necessary.

#### 4.2.8. Polysaccharide

Polysaccharides are naturally derived from plants and are macromolecular carbohydrates composed of monosaccharides linked by glycosidic bonds. Currently, various types of them have been identified, which can exert a broad range of biological activities [239]. Indeed, sulfated polysaccharides exhibit diverse biological activities, including antioxidant, neuroprotective, and anti-inflammatory effects [228,240]. These findings could align with the notions of their antinociceptive properties. Although evidence for their effectiveness in treating painful TMDs is currently lacking, preclinical studies have indicated the impacts of sulfated polysaccharides on craniofacial pain-like responses. For example, systemic administration of sulfated polysaccharides derived from red seaweed has been shown to reduce TMJ-evoked pain-like behaviors and enhance opioid function, as evidenced by increased levels of endorphins in the trigeminal subnucleus caudalis [83]. The reduction in TMJ formalin-evoked pain-like behaviors by polysaccharides derived from marine red algae is mediated through the modulatory effects of several receptor mechanisms, such as glutamatergic receptors [84]. A report, not cited in PubMed, revealed that sulfated polysaccharides derived from green seaweed could reduce mechanical hyper-nociception in the facial region 4 h after TMJ inflammation evoked by zymosan [85]. Despite a few preclinical reports, further human and preclinical studies are expected to assess the roles of polysaccharide-based treatments on craniofacial pain conditions. Additionally, polysaccharides derived from various plants have shown promise in preclinical studies for improving mental disorders such as anxiety and depression [239], which are risk factors for painful TMDs, thus supporting further exploration of their therapeutic potential. Collectively, it is too early to describe the definitive efficacy of polysaccharides on TMDs.

#### 4.2.9. Glucosamine

Glucosamine is an amino sugar and a prominent precursor in the biochemical synthesis of glycosylated proteins and lipids [241,242]. While glucosamine is an amino monosaccharide naturally biosynthesized in the human body, it is commonly extracted from the exoskeletons of crustaceans such as shrimp and crabs, as well as from animal cartilage and bone broth. It attaches to protein cores to form proteoglycans, which are essential components of the extracellular matrix in articular cartilage. The therapeutic potential of glucosamine in treating osteoarthritis has been well documented [243].

Indeed, as mentioned earlier, while randomized controlled trials assessing the effects of various nutraceuticals on painful TMDs have been scarce, this might not be the case for glucosamine. Several review articles have demonstrated that certain types of TMDs, particularly those involving degenerative joint diseases and pain, benefit from glucosamine administration [241,242,244]. The clinical efficacy of glucosamine in reducing pain associated with osteoarthritis of the TMJ appears to depend on the duration of administration, with benefits observed after more than 1–3 months of use [40,41,42,43,45,46]. However, a randomized clinical trial reported that glucosamine had a less beneficial effect on TMJ osteoarthritis pain compared to placebo controls [47].

Given the high prevalence of females with painful TMDs, a preclinical study using female rats might provide insights into the role of glucosamine in craniofacial pain; however, this study found that glucosamine caused no alterations in proinflammatory cytokine levels in both sham and ovariectomized females (OVX) [245]. These findings indicated that changes in sex hormones played a lesser role in those. Regardless of these findings, it is important to note that these reports did not focus on the therapeutic roles of glucosamine for myogenic TMDs.

Additionally, despite the unclear benefits of glucosamine alone, chicken bone broth, which contains glucosamine, exerted inhibitory effects on pain-like behaviors evoked by facial skin stimulation in a TMD model developed through prolonged jaw opening in rats [246]. These reports supported the notion that glucosamine might alleviate chronic pain in certain types of TMDs. Collectively, the efficacy of glucosamine on TMDs might be present in specific types of this condition. However, further clinical and preclinical evaluations and studies on other types of TMDs, like myogenic pain conditions, are necessary. 

#### 4.2.10. Palmitoylethanolamide

Palmitoylethanolamide (PEA) is a naturally occurring fatty acid amide found in various foods, such as tomatoes, soybeans, eggs, and broccoli. It is also synthesized in the body, including the brain, within the lipid bilayer [247]. Due to its antinociceptive, anti-inflammatory, and antioxidative effects [248], PEA has documented benefits in managing various chronic pain conditions. Various molecular mechanisms for its neural involvement have been demonstrated [249]. A randomized controlled trial revealed that the inhibitory effects of daily PEA administration on craniofacial pain, indicated by the maximum mouth opening, appeared to be greater than those of ibuprofen [48]. Additionally, PEA administration could reduce mechanical behavioral sensitivity in the facial region and glial activities in the trigeminal ganglion after the induction of inflammation in the TMJ [86]. Similarly, PEA might improve other types of chronic trigeminal pain conditions, including burning mouth syndrome and periodontal disease [247]. In the case of post-tooth extraction trigeminal neuropathy patients, PEA administration alone significantly alleviated pain as measured by a numeric rating scale [250]. Due to clinical and preclinical assessments of PEA’s efficacy seeming too premature, there is a need for more well-designed randomized controlled trials and animal studies to understand the roles of PEA on painful TMDs.

## 5. Limitations

While our current investigation shown above could allow us to advocate for clinical investigations into the beneficial role of nutraceuticals in managing TMDs, limitations are identified. First, a systematic review was not conducted. Indeed, due to the limited clinical and preclinical evidence available, this narrative review aims to elucidate current understandings of the relationships between nutrition and chronic craniofacial pain conditions rather than evaluating the efficacy of each intervention. Additionally, the literature search was confined to mainly Medline-PubMed and English-language sources, as outlined in the methods section. However, additional articles were retrieved through reference lists to avoid missing relevant studies. Second, our discussion relies heavily on preclinical evidence (38/50 articles), which could weaken the argument for the effectiveness of dietary and nutraceutical approaches from the clinical aspects. Attention should be paid to the preclinical models, as many experiments conducted pain assessments in the acute stages of pain. Therefore, additional approaches are crucial to assess the effects of dietary and nutraceutical interventions on chronic craniofacial nociception using models like psychological stress models [33,251]. Further, most preclinical studies developed TMD models using male rodents, while only two reports employed females [49,50]. Considering the predominance of females in TMDs, the conclusiveness of these statements based on the preclinical evidence was weakened. Despite those limitations, it is noteworthy that various models for painful TMDs have been well documented. These models share key features of painful TMDs, including behavioral characteristics and neural activities in the brain associated with craniofacial nociception, with or without therapeutic interventions [33]. This indicates that various preclinical models for TMDs have high translational value. Third, almost all preclinical studies reported predominantly positive findings, with little evidence of negative results. This might indicate publication bias, which could limit the comprehensiveness of our perspective. Therefore, future research should aim to standardize experimental protocols and improve reporting clarity to facilitate a more accurate and comprehensive evaluation of nutraceutical efficacy in managing craniofacial nociception. Fourth, while several mechanisms, such as the antioxidative and anti-inflammatory properties of nutraceuticals, were mentioned, detailed mechanisms of action at the molecular level and effective doses of each nutraceutical were not discussed. However, as mentioned earlier, these issues were beyond the scope of this investigation. Finally, randomized controlled trials involving dietary or nutraceutical interventions for managing TMDs were less common before 2015 [32]. These notions indicate that this area of research is still in its early stages and is actively developing. These limitations are important considerations for researchers to keep in mind in order to draw strong conclusions about the therapeutic roles of each nutraceutical on painful TMDs. 

## 6. Future Directions: An Alternative Diet—Rice-Fermented Food

**Figure 1:**
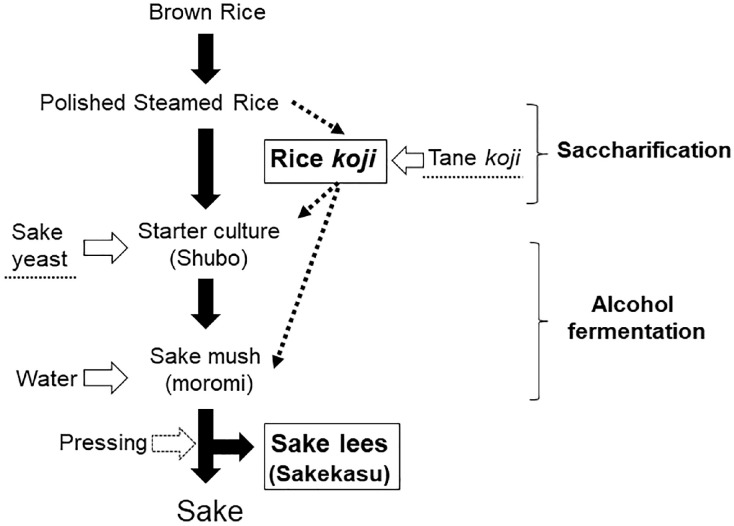
Japanese sake brewing process. Both Rice-koji and Sake Lees are byproducts during the processing.

Given the significant role of diet and nutrition in managing chronic pain, it is crucial to explore alternative dietary approaches. Recently, there has been increasing interest in the health-promoting effects of various fermented foods [252]. Among these, we would discuss the potential impacts of several rice-fermented foods, such as Sake lees and Rice-*koji*, on chronic pain. Those foods have been shown to contain various nutraceuticals. For example, Rice-*koji* contains vitamins B and E [253]. Another study has shown that it also contains phenolic acids, flavonoids, vitamin B3, and various amino acids such as tryptophan and GABA [254]. Sake lees consist of macro-nutrients like protein, carbohydrates, and fats [255], along with nutraceuticals including vitamins B3 and B6 [256]. Given the presence of these various nutraceuticals in rice-fermented foods, they hold promise for offering novel therapeutic benefits for painful TMDs. Although exhibiting significant geographical diversity [257], rice-fermented foods such as Sake lees and Rice-*koji* encompass a variety of traditional Japanese culinary delights renowned for their rich flavor and nutritional benefits [253,258]. Figure 1 illustrates the Japanese Sake brewing process, where the primary goal is to produce sake, the popular and traditional alcoholic beverage in Japan, alongside Rice-*koji* and Sake lees as byproducts, which contain various nutraceuticals. These products are bioactive, acting as probiotics that offer antioxidant and anti-inflammatory effects while also regulating various bodily functions [257,259]. Importantly, rice-fermented diets are known to be safe due to the absence of toxicity and adverse effects observed in in vitro and in vivo studies [260,261].

Our preclinical reports revealed that these rice-fermented foods could have modulatory roles on neural functions associated with nociception. First, daily administration of Sake lees attenuated pain-like hindpaw behaviors with the reduction of neural activities in the lumbar spinal dorsal horn in psychophysical stress rats [262]. Sake lees are a byproduct produced in large quantities during the brewing process of Japanese Sake (Figure 1). However, there are currently few effective methods for utilizing them. If Sake lees, rich in nutrients, could be consumed as functional food, it would reduce waste and align perfectly with the principles of the Sustainable Development Goals. Additionally, we reported an interesting finding [263]. An in vitro experiment demonstrated that Sake lees could induce functional changes in odontoblast-like cells, promoting their mineralization. Furthermore, an in vivo study revealed the facilitatory effects of Sake lees in producing reparative dentin-like hard tissues in the dental pulp of rats [263]. This evidence suggests that Sake lees might have potential applications in treating even toothache. Second, Japanese Sake displayed inhibitory effects on nociceptive neural activities in the trigeminal subnucleus caudalis region [264]. Of note, the results revealed that Sake effects are due to the constituents rather than the 15% ethanol found in Sake [264]. Third, daily consumption of Rice-*koji* prevented anxiety- and pain-like behaviors associated with psychological stress conditionings [260]. This study also revealed that ergothioneine, an antioxidant contained in Rice-*koji*, was well known for its regulatory roles in brain functions associated with pain [260]. While further research is needed to elucidate the precise mechanisms underlying these effects and assess their efficacy in clinical settings, our current studies propose that rice-fermented food could be the additional candidates that benefit from mitigating various chronic pain conditions, including TMDs.

## 7. Conclusions

Our review suggests that painful TMDs could influence dietary habits, leading to potential deficiencies in nutrients, yet the extent of this impact remains uncertain due to inconsistencies in research findings [108]. The multifactorial nature of TMDs, encompassing various pathologies, complicates efforts to draw definitive conclusions about their nutritional implications. However, given the multifactorial etiologies of TMDs, dietary improvements and nutraceutical approaches could be reasonable, as the regulatory effects of these interventions might address a wide range of bodily functions through their diverse constituents. This review discussed the roles of several nutraceuticals (Table 1) in chronic pain in the craniofacial tissues associated with TMDs, supported by preclinical evidence. Additionally, various rice-fermented foods might be suggested as supplementary nutritional approaches.

Emerging evidence indicates that supplementation with those nutraceuticals could hold promise in managing painful TMDs, aligning with observations in other chronic pain conditions. However, as mentioned in the section on limitations above, our understanding is limited by the scarcity of evidence from longitudinal and randomized controlled trials. Therefore, further preclinical investigations are required to clarify the therapeutic benefits of specific nutritional interventions to chronic pain in deep craniofacial tissues, which are prominently affected in TMDs.

Finally, a recent review highlighted the need for well-designed clinical trials based on dietary assessments and measurements capable of evaluating food quality and nutrient adequacy to assess the role of nutrition in TMDs [30]. This standpoint aligns with our ongoing inquiries. By advancing our understanding of the interplay between nutrition and craniofacial pain conditions, such as TMDs, through comprehensive research efforts, we can pave the way for personalized therapeutic strategies that enhance the quality of life for individuals affected by these conditions.

## Figures and Tables

**Table 1:** List of nutraceuticals. Individual nutraceuticals discussed are shown in parentheses.

Nutraceuticals	
Vitamin Mineral Polyunsaturated fatty acids Polyphenols Isoprenoids Carotenoid Lectin Polysaccharide Glucosamine Palmitoylethanolamide	Vitamin D, Vitamin B, Vitamin C Magnesium, Zinc, Strontium, Sulfur Omega-3 fatty acids, Docosahexaenoic acid Quercetin, (-)-epigallocatechin-3-gallate, Resveratrol, Curcumin Limonene, Citral Crocin, Lutein

**Table 2:** List of keywords employed in this study. For searching clinical and preclinical studies, the keyword was selected from categories “a” or “b” and “c”, respectively.

Category	Keywords
a. Clinical	“temporomandibular disorder” OR “TMD” OR “temporomandibular joint” OR “TMJ” OR “masseter muscle” OR “orofacial pain” OR “patient” OR “clinical”
b. Preclinical	“craniofacial tissue” OR “animal model” OR “Complete Freund’s Adjuvant” OR “CFA” OR “formalin” OR “mice” OR “rat” OR “nociception” OR “preclinical model”
c. Exposure	“antioxidant” OR “diet” OR “dietary” OR “dietary supplement” OR “supplement” OR “fatty acid” OR “fiber” OR “food” OR “mineral” OR “natural product” OR “nutraceutical” OR “nutrition” OR “supplement” OR “phytochemical” OR “polyphenol” OR “vitamin” OR “fruits” OR “vegetable” OR “isoprenoid” OR “carotenoid” OR “lectin” OR “polysaccharide” OR “glucosamine”

**Table 3:** Clinical studies evaluating the effects of nutraceutical interventions in Temporomandibular Disorders.

Authors	Pain Condition	Study Duration	Interventions	Groups	Outcomes
Vitamins					
Gupta et al., 2022, India [37]	Axis I group II TMDs with vitamin D levels < 30 ng/mL.	3 months	Vitamin D tablets 60,000 IU once a week for eight weeks.	1. Splint alone 2. Splint + Vitamin D supplement	In TMD patients with vitamin D deficiency, a significant difference was seen in VAS score and maximum mouth opening between the splint with vitamin D supplementation and the splint with a placebo drug.
Reis et al., 2023, Brazil [38]	Chronic myofascial pain and arthralgia.	1 month	Methylcobalamin (B12) 1000 μg/day, orally.	1. Laser and B12 placebo 2. Effective laser + B12 placebo 3. Effective laser + B12	Vitamin B12 facilitates the inhibitory effects of laserpuncture in treating painful TMDs.
Minerals					
Refahee et al., 2022, Egypt [44]	Myofascial pain and trigger points in the masseter muscle.	6 months	Magnesium sulfate (MS, 0.41 mMol/mL) 2 mL, trigger point injection.	1. Saline 2. Magnesium sulfate	MS reduced the facial pain scores, and the maximum mouth opening distance was higher up to 3 months in the MS than in the saline group.
Kiliç, 2021, Turkey [39]	Temporomandibular joint osteoarthritis (TMJ OA).	12 months	GCM supplementation, containing 750 mg GH, 600 mg chondroitin sulfate, and 350 mg MSM at 2 × 1 dosage daily for 3 months.	1. arthrocentesis plus intraarticular hyaluronic acid (HA) injection only 2. arthrocentesis plus intraarticular HA injection followed by 3 months of GCM	GCM supplementation after arthro-centesis plus intraarticular hyaluronic acid injection produced no additional clinical benefits or improvements for patients with TMJ-OA compared with arthrocentesis plus intraarticular hyaluronic acid injection alone.
Glucosamine					
Thie et al., 2001, Canada [45]	Degenerative joint Disease of TMJ.	4 months	Glucosamine sulfate (GS) 500 mg tid for 90 days, orally.	1. Ibuprofen 2. GS	GS decreased TMJ pain compared with ibuprofen administrations.
Damlar et al., 2014, Turkey [40]	Internal derangements of TMJ	2 months	1500 mg glucosamine and 1200 mg chondroitin sulfate (CS) /day, orally.	1. Tramadol HCl 2. glucosamine and chondroitin sulfate	A combination of glucosamine and chondroitin sulfate reduced pain compared with the tramadol group.
Haghighat et al., 2013, Iran [46]	Painful TMJ, TMJ crepitation or limitation of mouth opening.	3 months	Glucosamine sulfate (GS) 1500 mg/day, orally.	1. Ibuprofen 2. GS	GS improved craniofacial pain and mandibular opening distance compared to baseline and showed more post-treatment improvement when compared with ibuprofen.
Cahlin et al., 2011, Sweden [47]	TMJ OA	6 weeks	Glucosamine sulfate (GS) 1200 mg/day, orally.	1. Placebo drug 2. GS	GS showed less beneficial effects compared with the placebo group in reducing pain associated with TMJ osteoarthritis.
Nguyen et al., 2016, USA [41]	Capsulitis, disk displacement, disk dislocation, or painful osteoarthritis of TMJ	12 weeks	1500 mg of glucosamine hydrochloride (GH) and 1200 mg of chondroitin sulfate (CS)/day, orally.	1. Placebo drug 2. GH + CS	GH-CS reduced craniofacial pain compared with the placebo group.
Cen et al., 2017 [42]	TMJ OA	1 year	Two tablets of 240 mg glucosamine hydrochloride (GH) tid for 3 months, orally	1. Placebo + HA injection 2. GH + HA injection	GH with HA injection decreased craniofacial pain at 1 month and one year follow-up compared to baseline. One year later, pain score was reduced, and IL-6 and IL-1β levels were lower in group GH + HA than in group placebo + HA.
Yang et al., 2018, China [43]	TMJ OA	1 year	Glucosamine hydrochloride (GH) 1.44 g/day for 3 months, orally.	1. Placebo + hyaluronate sodium injection 2. GH + hyaluronate sodium injection	GH and hyaluronate sodium injection improved maximal mouth opening distance and facial pain intensity compared to the placebo + hyaluronate sodium injection group in the long-term follow-up.
Palmitoylethanolamide					
Marini et al., 2012, Italy [48]	TMJ OA or arthralgia	2 weeks	Palmitoylethanolamide (PEA) 300 mg in the morning and 600 mg in the evening for 7 days and then 300 mg twice a day for 7 more days, orally.	1. Ibuprofen 2. PEA	PEA improved pain related to maximum mouth opening compared with the ibuprofen-treated group.

**Table 4:** Preclinical studies evaluating the effectiveness of nutraceutical interventions in TMD models.

Authors	Model	Interventions	Groups	Outcomes
Vitamins				
Erfanparast et al., 2014 [58]	Male rats, formalin test	Vitamin B12, 1, 2, and 4 mg/kg, i.p. or peripheral (2.5, 5 and 10 µg/rat) injections.	1. Saline 2. Diclofenac 3. Vitamin B12 4. Vitamin B12 + Diclofenac	Vitamin B12 reduced facial pain-like behavior. Co-treatments with vitamin B12 and diclofenac facilitated antinociceptive effects.
Erfanparast et al., 2017 [59]	Male rats, formalin test	Vitamin B12, 0.5 µL intra-hippocampal injection.	1. Saline 2. Naloxone 3. Vitamin B12 4. Vitamin B12 +Naloxone	Vitamin B12 reduced facial pain-like behaviors associated with laserpuncture intervention.
Minerals				
Cavalcante et al, 2013 [60]	Male rats, carrageenan-induced TMJ inflammation	Magnesium chloride (MgCl_2_), 10, 30, and 90 mg kg/day, orally.	1. Naive (no carrageenan) 2. Carrageenan + Vehicle 3. Carrageenan + MgCl_2_	MgCl_2_ reduced facial pain-like behaviors evoked by mechanical threshold.
Srebro et al., 2018 [61]	Male rats, formalin test	Magnesium sulfate (MS), 0.005–45 mg/kg, s.c.	1. Naïve (no formalin) 2. Formalin + Vehicle 3. Formalin + MS	MS reduced facial pain-like behaviors.
Srebro et al., 2023 [62]	Male rats, formalin test	Magnesium sulfate (MS), 5, 15 mg/kg, s.c.	1. Saline 2. Cromoglycate 3. MS 4. MS + Cromoglycate	MS reduced formalin-induced facial pain-like behaviors.
Alves et al., 2017 [63]	Male rats, zymosan-induced TMJ inflammation	Strontium, 0.5, 5, or 50 mg/kg, orally.	1. Naïve (no zymosan) 2. Zymosan 3. Zymosan + Indomethacin 4. Zymosan + Strontium ranelate	Strontium reduced the facial pain-like behaviors evoked by mechanical threshold.
Fatty acids				
Marana et al, 2022 [49]	Female rats, type II bovine collagen (CII) +CFA-induced rheumatoid arthritis (RA)	Omega-3 FAs, 300 mg/kg/day, orally.	1. Naïve (non-RA) 2. ovariectomized rats (OVX) 3. OVX + RA 4. OVX + Omega-3 FAs	Omega-3 FAs reduced TMJ damage and increased proinflammatory cytokine levels.
Ceotto et al., 2022 [64]	Male rats, CII +CFA-induced RA	Omega-3 FAs, 85 mg/kg/day, orally.	1. Naïve (non-RA) 2. RA 3. RA + Omega-3 FAs 4. RA + Omega-3 FAs +Aspirin	Omega-3 FAs reduced TMJ damage identified and increased various cytokines.
Barbin et al., 2020 [65]	Rats, Sex: not described, CFA-induced facial inflammation	Omega-3 FAs, 300 mg/kg/day, orally.	1. Naïve (no CFA) 2. CFA + Saline 3. CFA + dexamethasone 4. CFA + Omega-3 FAs	Omega-3 FAs reduced TMJ damage identified by histo-morphometric analysis. Differences in the level of IL-1 beta, TNF-alpha, and IL-10 between control and CFA groups were found.
Nakazaki et al., 2018 [66]	Male rats, CFA-induced TMJ inflammation)	Docosahexaenoic acid (DHA), 328 mg/kg, i.p.	1. Naïve (no CFA) 2. CFA + Saline 3. CFA + DHA	DHA reduced mechanical behavioral sensitivities and neural activities in the trigeminal subnucleus caudalis.
Polyphenols				
Sashide et al., 2024 [51]	Male rats, CFA-induced facial inflammation	Quercetin, 1–10 mM, local injection.	1. Before quercetin injection 2. After quercetin injection	Quercetin reduced facial stimulation-evoked neural activities in the trigeminal ganglion (TG).
Toyota et al., 2023 [52]	Male rats, naïve	Quercetin, 1, 10 mM, local injection.	1. Before quercetin injection 2. After quercetin injection	Quercetin reduced facial stimulation-evoked nociceptive neural activities in the TG.
Liu et al., 2024 [53]	Male rats, LPS into the TG	Quercetin, 10 mM, trigeminal ganglion (TG) injection.	1. Naïve (no LPS) 2. LPS + Saline 3. LPS + Quercetin	Quercetin reduced mechanical hyperalgesia-like behaviors in rats with the intra-TG injection of LPS.
Itou et al., 2022 [54]	Male rats, CFA-induced whisker pad inflammation	Quercetin, 50 mg/kg, i.p.	1. Naïve (no CFA) 2. CFA + Saline 3. CFA + Diclofenac 4. CFA + Quercetin	Quercetin reduced mechanical hyperalgesia-like behaviors and neural activities in the trigeminal subnucleus caudalis.
Uchino et al., 2023 [55]	Male rats, naïve	(-)-epigallocatechin-3-gallate (EGCG), 1, 10 mM, local injection.	1. Before EGCG injection 2. After EGCG injection	EGCG reduced neural activities in the trigeminal subnucleus caudalis region evoked by mechanical stimulation to the facial skin.
Shimazu et al., 2016 [56]	Male rats, naïve	Resveratrol (RSV), 1–10 mM, local injection.	1. Before RSV injection 2. After RSV injection	Resveratrol suppresses the excitability of the trigeminal subnucleus caudalis neurons.
Ma et al., 2020 [67]	Male rats, CFA-induced facial inflammation	Resveratrol (RSV), 40 mg/kg or 80 mg/kg, i.p.	1. Naïve (no CFA) + Dimethyl sulfoxide (DMSO) 2. Naïve (no CFA) + RSV 3. CFA + DSMO 4. CFA + RSV	Resveratrol increased the CFA-decreased mechanical withdrawal threshold.
Takehana et al., 2017 [57]	Male rats, naïve	Resveratrol, 2 mg/kg, i.v.	1. Before RSV injection 2. After RSV injection	Resveratrol reduced neurotransmission of the trigeminal nucleus caudalis neurons evoked by mechanical stimulation.
Mittal et al., 2009 [50]	Male and female rats, formalin test	Curcumin, 25, 50, 100, 200, 400, and 600 mg/kg, i.p.	1. DMSO 2. Curcumin 3. Diclofenac + Curcumin	Curcumin reduced facial pain-like behaviors.
Luca et al., 2014 [68]	Male mice, formalin test	Curcumin, 120 mg/kg, orally.	1. Vehicle 2. Curcumin	Curcumin reduced facial pain-like behaviors.
Wu et al., 2017 [69]	Male rats, formalin test	Curcumin, 50 mg/kg, i.p.	1. Naïve (no formalin) 2. Formalin 3. Formalin + Amiloride 4. Formalin + Curcumin	Curcumin reduced facial pain-like behaviors.
Yeon et al., 2010 [70]	Male rats, capsaicin test	Curcumin, 5, 25, or 50 mg/kg, i.p.	1. Naïve (no capsaicin) 2. Naïve (no capsaicin) + Curcumin 3. Capsaicin + Vehicle 4. Capsaicin + Curcumin	Curcumin reduced capsaicin-induced thermal hyperalgesia-like behaviors in rats.
Isoprenoids				
Pereira et al., 2022 [71]	Male rats, formalin test	Limonene (LIM), 50 mg/kg, orally.	1. Naïve (no formalin) 2. Formalin 3. Formalin + Morphine 4. Formalin + LIM 5. Formalin + LIM/HPβCD	Limonene reduced TMJ pain-like behaviors.
Santos et al., 2023 [72]	Male rats, formalin test or CFA-induced TMJ inflammation	Citral, 100 and 300 mg/kg, orally.	1. Formalin + Tween80 2. Formalin + Citral 1. Naïve (no CFA) + Tween80 2. CFA + Tween80 3. CFA + Citral	Citral reduced formalin-induced orofacial nociceptive behavior and mechanical hyper-nociception in CFA-induced TMJ inflammation.
Santos et al., 2022 [73]	Male mice and rats, formalin, mustard oil, cinnamaldehyde, menthol, and capsaicin tests	Citral, 0.1, 0.3, or 1.0 mg/kg, orally.	1. Naïve (non-inflamed) 2. Inflamed + vehicle 3. Inflamed + Citral	Citral reduced TMJ- or masseter muscle-evoked pain-like behaviors via TRPV1, TRPM3, and TRPM8 mechanisms.
Carotenoids				
Tamaddonfard et al., 2015 [74]	Male rats, capsaicin test	Crocin, 10 and 40 µg/rat, Intra-fourth ventricle injection.	1. Capsaicin + Saline 2. Capsaicin + Morphine 3. Capsaicin + Crocin 4. Capsaicin + Morphine + Crocin	Intra-fourth ventricle administration of crocin reduced facial pain-like behavior independent of opioid mechanisms.
Shimazu et al., 2019 [75]	Male rats, mustard oil-evoked inflammation	Lutein, 10 mg/kg, i.p.	1. Naïve (no mustard oil) 2. Mustard oil 3. Mustard oil + Lutein	Lutein reduced facial pain-like behaviors and c-Fos responses in the trigeminal caudalis and upper cervical dorsal horn.
Syoji et al., 2018 [76]	Male rats, CFA-induced inflammation	Lutein, 10 mg/kg, i.p.	1. Naïve (no CFA) 2. CFA 3. CFA + Lutein	Lutein reduced mechanical hyperalgesia-like behaviors via the Cox-2 signaling cascade.
Lectins				
Rivanor et al., 2018 [77]	Male rats, formalin or carrageenan or capsaicin test	Lectin, 0. 1, 1, or 10 mg/kg, i.v.	1. Naïve (non-inflamed) 2. Inflamed + Saline 3. Inflamed + Lectin	Lectin from green seaweed Caulerpa cupressoides reduced orofacial pain-like behaviors.
Rivanor et al., 2014 [78]	Male rats, Zymosan	Lectin, 0.1, 1, or 10 mg/kg, i.v.	1. Naïve (no zymosan) 2. Zymosan + Saline 3. Zymosan + Indomethacin 4. Zymosan + Lectin	Lectin from the green seaweed Caulerpa cupressoides reduced mechanical hyper-nociception.
Leite et al., 2022 [79]	Mice and rats, Sex: not described, capsaicin, formalin test	Lectin, 0.25, 0.5, 1, 10 mL/kg, i.p.	1. Naïve (non-inflamed) 2. Inflamed + Saline 3. Inflamed + Lectin	Parkia platycephala lectin reduced orofacial pain-like behavior.
Alves et al., 2018 [80]	Male rats, formalin test	Lectin, 0.001–0.1 mg/kg, i.v.	1. Naïve (no formalin) 2. Formalin 3. Formalin + Morphine 4. Formalin + Lectin	Lectin from Abelmoschus esculentus reduced orofacial pain-like behavior.
Freitas et al., 2016 [81]	Male rats, zymosan-induced TMJ inflammation	Lectin, 0.01–1 mg/kg, i.v.	1. Naïve (no zymosan) 2. Zymosan + Saline 3. Zymosan + Indomethacin 4. Zymosan + Lectin	Lectin from Abelmoschus esculentus reduced mechanical hyperalgesia-like behaviors.
Damasceno et al., 2016 [82]	Mice and rats, Sex: not described, formalin, capsaicin, glutamate test	Frutalin, 0.25, 0.5, or 1 mg/kg, i.p.	1. Naïve (non-inflamed) 2. Inflamed + Saline 3. Inflamed + Frutalin	Frutalin reduced facial pain-like behaviors via nitrogenic oxide mechanisms.
Polysaccharides				
Araújo et al., 2017 [83]	Male rats, formalin test	Sulfated polysaccharide (SP), 0.03, 0.3 or 3.0 mg/kg, s.c.	1. Naïve (no formalin) 2. Formalin 3. Formalin + Sulfated polysaccharide	SP from the red seaweed Solieria filiformis reduced facial pain-like behaviors, inhibited the plasma extravasation and inflammatory cytokines release, and increased the β-endorphin release in the trigeminal caudalis.
Souza et al., 2019 [84]	Male mice or rats, formalin test	Sulfated polysaccharide (SP), 5, 10 mg/kg, orally	1. Naïve (no formalin) 2. Formalin 3. Formalin + Sulfated polysaccharide	SP from the marine algae Hypnea pseudomusciformis reduced orofacial pain-like behaviors.
Rodrigues et al., 2014 [85]	Male rats, zymosan-evoked TMJ inflammation	Sulfated polysaccharide (SP), 1, 3 and 9 mg/kg, s.c.	1. Naïve (no zymosan) 2. Zymosan + Saline 3. Zymosan + Indomethacin 4. Zymosan + Sulfated polysaccharide	SP from the green seaweed Caulerpa cupressoides var. lycopodium reduced mechanical hyperalgesia-like behaviors.
Palmitoylethanolamide				
Bartolucci et al., 2018 [86]	Male rats, CFA-induced facial inflammation	Palmitoylethanolamide (PEA), 10 mg/kg, i.p.	1. Naïve (no CFA) 2. Naïve (no CFA) + PEA 3. CFA + Vehicle 4. CFA + PEA	PEA reduced facial hyperalgesia-like behaviors after TMJ inflammation.

Abbreviations: i.p., intraperitoneal injection; i.v., intravenous injection; s.c., subcutaneous injection; TG, trigeminal ganglion.

## Data Availability

Data sharing is not applicable to this article as no new data were created or analyzed in this study.

## Supplementary Materials

The following supporting information can be downloaded at: https://www.mdpi.com/article/10.3390/nu16172868/s1. Table S1: Risk of bias assessment for clinical studies; Table S2: Risk of bias assessment for preclinical studies.
