# Supplementary material for: Nutritional Strategies for Chronic Craniofacial Pain and Temporomandibular Disorders: Current Clinical and Preclinical Insights
Source: Nutrients. 2024 Aug 27;16(17):2868. doi: 10.3390/nu16172868 (PMC11397166; doi:10.3390/nu16172868)
Supplement: Supplementary file 1 [file nutrients-16-02868-s001.zip › Table S1.pdf]

Table S1. Risk of bias assessment for clinical studies.

Studies	Type of intervention	1	2	3	4	5	6	7
Gupta et al., 2022 [37]	Vitamin D	+	+	+	+	+	+	+
Reis et al., 2023 [38]	Vitamin B12	+	?	+	+	+	+	+
Refahee et al., 2022 [44]	Magnesium	+	+	+	+	+	+	+
Kiliç, 2021 [39]	Glucosamine	?	?	+	+	?	?	+
Thie et al., 2001 [45]	Glucosamine	+	+	+	+	+	+	+
Damlar et al., 2014 [40]	Glucosamine	?	+	+	+	+	+	+
Haghighat et al., 2013 [46]	Glucosamine	?	?	+	+	?	?	+
Cahlin et al., 2011 [47]	Glucosamine	+	+	+	+	+	+	+
Nguyen et al., 2016 [41]	Glucosamine	+	+	+	+	+	+	+
Cen et al., 2017 [42]	Glucosamine	+	+	+	+	+	+	+
Yang et al., 2018 [43]	Glucosamine	+	+	+	+	+	+	+
Marini et al., 2012 [48]	Palmitoylethanolamide	+	?	+	+	+	+	+

1: random sequence generation; 2: allocation concealment; 3: selective reporting; 4: other bias; 5: blinding of participants and personnel; 6: blinding of outcome assessment; 7: incomplete outcome data. +: low risk of bias; ?: unclear risk; −: high risk of bias.
