# Supplementary material for: Nutritional Strategies for Chronic Craniofacial Pain and Temporomandibular Disorders: Current Clinical and Preclinical Insights
Source: Nutrients. 2024 Aug 27;16(17):2868. doi: 10.3390/nu16172868 (PMC11397166; doi:10.3390/nu16172868)
Supplement: Supplementary file 1 [file nutrients-16-02868-s001.zip › Table S2.pdf]

Table S2. Risk of bias assessment for preclinical studies

Studies	intervention	1	2	3	4	5	6	7	8	9	10
Erfanparast et al., 2014 [58]	Vitamin B12	?	?	?	?	?	?	?	+	+	+
Erfanparast et al., 2017 [59]	Vitamin B12	?	?	?	?	?	?	?	+	+	+
Cavalcante et at, 2013 [60]	Magnesium	+	?	?	?	?	?	+	−	+	+
Srebro et al., 2017 [61]	Magnesium	?	?	?	?	?	?	?	+	+	+
Srebro et al., 2023 [62]	Magnesium	?	?	?	?	?	?	+	+	+	+
Alves et al., 2017 [63]	Strontium	?	?	?	?	?	?	+	?	+	+
Marana et al, 2022 [49]	Omega-3 Fatty acids	?	+	?	?	?	?	?	+	+	+
Ceotto et al., 2022 [64]	Omega-3 Fatty acids	?	+	?	?	?	?	+	+	+	+
Barbin et al., 2020 [65]	Omega-3 Fatty acids	?	?	?	?	?	?	?	?	+	+
Nakazaki et al., 2018 [66]	Docosahexaenoic Acid	?	?	?	?	?	?	?	+	+	+
Sashide et al., 2024 [51]	Quercetin	?	?	?	?	?	?	?	+	+	+
Toyota et al., 2023 [52]	Quercetin	?	?	?	?	?	?	?	+	+	+
Liu et al., 2024 [53]	Quercetin	+	?	?	?	+	?	+	+	+	+
Itou et al., 2022 [54]	Quercetin	?	?	?	?	?	?	?	+	+	+
Uchino et al., 2023 [55]	(-)-epigallocatechin-3-gallate	?	?	?	?	?	?	?	?	+	+
Shimazu et al., 2016 [56]	Resveratrol	?	?	?	?	?	?	?	+	+	+
Ma et al., 2020 [67]	Resveratrol	?	?	?	?	?	?	?	−	+	+
Takehana et al., 2017 [57]	Resveratrol	?	?	?	?	?	?	?	−	+	+
Mittal et a., 2009 [50]	Curcumin	?	−	?	?	?	?	?	?	+	+
Luca et al., 2014 [68]	Curcumin	?	?	?	?	?	?	?	?	+	+
Wu et al., 2016 [69]	Curcumin	?	?	?	?	?	?	+	−	+	+
Yeon et al., 2010 [70]	Curcumin	?	?	?	?	?	?	+	+	+	+
Pereira et al., 2022 [71]	Limonene	?	+	?	?	?	?	+	?	+	+
Santos et al., 2023 [72]	Citral	?	?	?	?	?	?	+	+	+	+
Santos et al., 2022 [73]	Citral	?	+	?	?	?	?	?	+	+	+
Tamaddonfard et al., 2015 [74]	Crocin	?	?	?	?	?	?	+	+	+	+
Shimazu et al., 2019 [75]	Lutein	?	?	?	?	?	?	?	−	+	+
Syoji et al., 2018 [76]	Lutein	?	?	?	?	+	?	+	+	+	+
Rivanor et al., 2018 [77]	Lectin	?	?	?	?	+	?	+	+	+	+
Rivanor et al., 2014 [78]	Lectin	?	?	?	?	?	?	?	+	+	+
Leite et al., 2022 [79]	Lectin	?	?	?	?	?	?	?	?	+	+
Alves et al., 2018 [80]	Lectin	?	?	?	?	?	?	?	−	+	+
Freitas et al., 2016 [81]	Lectin	?	?	?	?	?	?	+	+	+	+
Damasceno et al., 2016 [82]	Frutalin	?	?	?	?	?	?	?	+	+	+
Araújo et al., 2017 [83]	polysaccharide	?	?	?	?	?	?	?	?	+	+
Souza et al., 2018 [84]	polysaccharide	?	?	?	?	?	?	?	+	+	+
Rodrigues et al., 2014 [85]	polysaccharide	?	?	?	?	?	?	?	+	+	+
Bartolucci et al., 2018 [86]	Palmitoylethanolamide	+	?	?	?	?	?	?	+	+	+

1: sequence generation; 2: baseline characteristics; 3: allocation concealment; 4: random housing; 5: blinding of participants and personnel; 6: random outcome assessment; 7: blinding of outcome assessment; 8: incomplete outcome data; 9: selective outcome reporting; 10: other sources of bias. +: yes (low risk of bias); ?: unclear; −: no (high risk of bias).
